# Emerging Role of Immunosuppression in Diseases Induced by Micro- and Nano-Particles: Time to Revisit the Exclusive Inflammatory Scenario

**DOI:** 10.3389/fimmu.2018.02364

**Published:** 2018-11-19

**Authors:** François Huaux

**Affiliations:** Louvain Centre for Toxicology and Applied Pharmacology, Institut de Recherche Experimentale et Clinique, Université catholique de Louvain, Brussels, Belgium

**Keywords:** immunosuppression, inflammation, TGF-β, IL-10, immunosuppressive lymphocytes, immunosuppressive myeloid cells

## Abstract

Fibrosis, cancer, and autoimmunity developing upon particle exposure have been exclusively linked with uncontrolled inflammatory processes. The critical role of inflammation is now challenged by several contradictory observations indicating that the emergence of these chronic disorders may result from non-inflammatory events. A growing number of studies reveals that micro- and nano-particles can cause exaggerated and persistent immunosuppression characterized by the release of potent anti-inflammatory cytokines (IL-10 and TGF-β), and the recruitment of major regulatory immune cells (M2 macrophages, T and B regs, and MDSC). This persistent immunosuppressive environment is initially established to limit early inflammation but contributes later to fibrosis, cancer, and infection. Immunosuppression promotes fibroblast proliferation and matrix element synthesis and subverts innate and adaptive immune surveillance against tumor cells and microorganisms. This review details the contribution of immunosuppressive cells and their derived immunoregulatory mediators and delineates the mutual role of inflammatory vs. immunosuppressive mechanisms in the pathogenesis of chronic diseases induced by particles. The consideration of these new results explains how particle-related diseases can develop independently of chronic inflammation, enriches current bioassays predicting particle toxicity and suggests new clinical strategies for treating patients affected by particle-associated diseases.

## Micro- and nano-particles in the twenty-first century

The toxicity of inhaled particles and the subsequent development of health diseases including fibrosis, cancer and autoimmunity are recognized since several centuries (1). Unfortunately, the available scientific knowledge is not yet adequate for monitoring particle exposure-related health problems, delivering treatment to affected patients, and devising appropriate early recognition of reactive particles and individuals at risk (2–4). For example, although silicosis has been known for decades, exposure to dusts containing free crystalline silica remains uncontrolled in countless workplaces throughout the world and clinical cases of chronic diseases caused by inhaled particles are still frequent (5–7). Despite active research, the highly lethal disorders caused by silica inhalation continue to pose major clinical challenges because there are refractory to current therapeutic strategies (8). In addition, clinical detection of silica-associated lung diseases is currently dependent on radiological and lung function tests, which are both late manifestations of diseases (8). Therefore, effective therapeutic regimen and early disease detection have yet to be identified and developed. This problematic is not limited to naturally occurring mineral dusts. The recent ability to manipulate the structure and the morphology of particles, allowing the production of tailor-made dusts with specific size, form and function opens very attractive avenues in nanobiotechnologies and industries. However, for the successful application of these new materials now largely designed and produced worldwide, it is becoming evident that the biological fate and potential toxicity of these particles have to be actively determined (9). Hence, it is becoming vital to understand how complex pathologies (fibrosis, cancer, and autoimmune disorders) are induced by inhaled particles in order to reduce their impacts on human health.

## Inflammation as the driving force

The fundamental concept, usually put forward to explain the pathogenesis of particle-induced fibrosis, cancer, and autoimmune diseases, is relatively simple. An unresolved and chronic inflammatory process, characterized by marked inflammatory cell accumulation and sustained release of inflammatory molecules, damages tissues, orchestrates accumulation of mesenchymal cells and their matrix protein products, transforms normal cells to tumor cells or activates adaptive immune responses to self-antigens (10).

**Figure 1:**
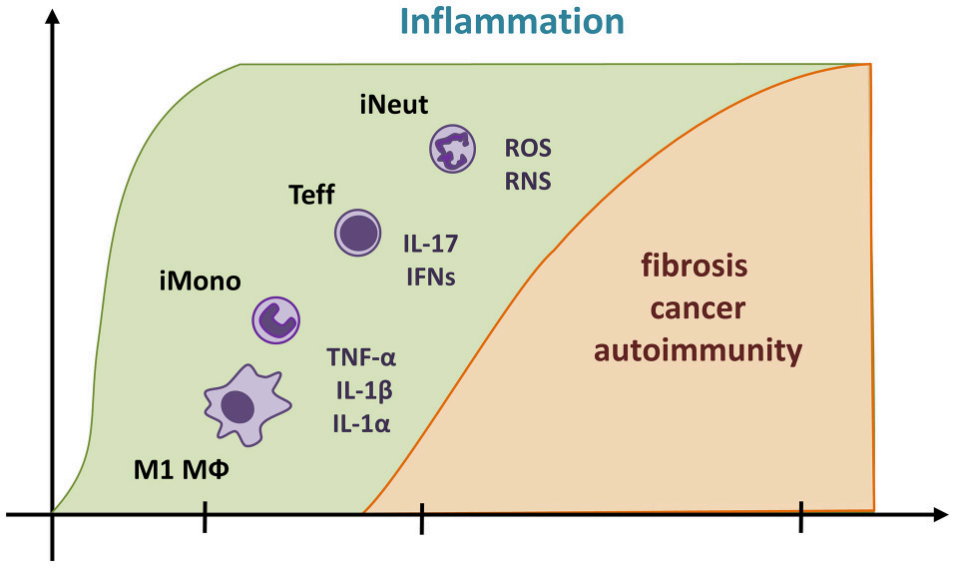
Predominant unresolved inflammation contributes in the development of chronic diseases related to particle exposure. The pathological pathway classically described suggests that a predominant and persistent inflammatory process (in green) orchestrates fibrosis, cancer, and autoimmune diseases caused by particles (in yellow). Reactive particles induce an inflammatory cascade, which implies TNF-α, IL-1 α, and β, IFNs (IFN-γ and β), IL-17, and free radicals (ROS and RNS) and precedes the influx of inflammatory macrophages (M1 Mϕ), inflammatory myeloid cells (iMono, inflammatory monocytes; iNeut, inflammatory neutrophils), and effector T lymphocytes (T eff, comprising Th1, γδ T cells, and Th17). When immunosuppressive activities are insufficient, these pro-inflammatory mediators and cells persist and result in to uncontrolled cycle of injury. Pro-inflammatory cytokines are also considered as potent polypeptide growth factors for mesenchymal cells and ultimately induce fibrosis. Sustained production of free radicals induces irreversible DNA damage and results in carcinogenesis. Constant tissue damage and inflammation activate adaptive immunity, autoantibody production and autoimmunity.

A precise cascade of cellular and molecular inflammatory events has been proposed from an extensive and growing number of data in human and animal exposed to particles (Figure 1). First, sentinel (alveolar and interstitial macrophages) and resident cells (epithelial cells and fibroblasts) actively recognize particles through sophisticated innate immune platforms (11, 12). These activated cells release various inflammatory mediators such as cytokines, chemokines, eicosanoids, and free radicals (13–16). These mediators induce a marked and persistent recruitment of inflammatory myeloid or lymphoid cells (inflammatory M1 macrophages; neutrophils, monocytes, and effector T lymphocytes) that results in tissue damages. Persistent inflammation-induced injury is subsequently followed by an exaggerated reparative phase in which polypeptide growth factors produced by inflammatory cells stimulate non-controlled fibroblast recruitment, proliferation, and matrix protein production (1). Released reactive oxygen or nitrogen species (ROS and RNS) play a major role in the genotoxic activity of particles. Under inflammatory conditions, free radicals generated from the particle itself or inflammatory cells induce DNA damage in target cell populations and participate in the carcinogenic process (17). Again, tissue damage and inflammation associated to inhaled particles are the suspected pathological pathway that leads to immune abnormalities, adaptive immunity activation, tolerance breaking, antinuclear autoantibody production and finally systemic autoimmune diseases including lupus and arthritis (10).

A myriad of inflammatory mediators accompanying inflammation are linked to scar formation, cell transformation, and autoimmunity but only few of them are definitively identified as important in the pathological processes. Among those, the master pro-inflammatory cytokines TNF-α, IL-1 (mainly produced by M1 macrophages), type I and II IFN and IL-17 (from effector T lymphocytes as Th1, γδ T cells, and Th17) are essential in the pathogenesis of particle-induced diseases (10, 18–20, 21). These inflammatory mediators are responsible of leukocyte influx in damaged tissues and orchestrate aggregation of mature phagocytic macrophages and granuloma formation (22, 23). In addition, they promote fibroblast activation and differentiation (24), epithelial or mesothelial cell transformation (13, 25) and immune responses to self-antigens (26–28). Inflammatory cell accumulation, collagen deposition, or malignant mesothelioma in silica- or asbestos-treated mice was markedly decreased by suppressing genetically or pharmacologically these key inflammatory mediators (13, 29). Increased levels of TNF-α, IL-1, IFN-γ, and IL-17 have been observed in human under conditions of developing silicosis, asbestosis or mesothelioma (30–33). These findings convincingly indicate a causal relationship between excessive or maintained accumulation of pro-inflammatory cells and cytokines and the establishment of particle-induced chronic diseases.

## Recent clinical and experimental findings that challenge the inflammatory dogma

Current treatment modalities for treating patients developing particle-related diseases have been based on the assumption that these disorders are exclusively related to chronic inflammation diseases. However, it is becoming evident that most available anti-inflammatory drugs (34, 35) or cytokine inhibitor therapy (36) are not effective for particle-exposed patients. Human validation studies have also failed to confirm the use of inflammatory mediators as biomarkers for univocally detecting the health effects of particles (37).

Besides the strong evidence of a major role of inflammation in particle-treated animals, several experimental studies did not find an evident association between inflammatory responses and experimental diseases and, thus, have also challenged this inflammatory dogma. Several studies, conducted in mice, reported that exacerbated lung inflammation is not always accompanied by a subsequent increase of fibrosis after silica or carbon nanotubes (CNT) treatment (38, 39). Inversely, limited inflammation can be associated to robust silica-induced long-term lung responses (40). More precisely, it has been found that a progressive neutrophilic inflammatory process and a sustained release of pro-inflammatory cytokines did not accompany instillation of silica or CNT in mice. In this case, a mild and non-progressive pulmonary inflammation and a transient production of pro-inflammatory cytokines such TNF-α and IL-1β was instead observed (22, 41–43). Additionally, these inflammatory components were dispensable for the development of particles-induced fibrogenesis since genetic deficiencies of these master pro-inflammatory cytokines did not completely limit lung fibrosis while they were efficient to abrogate inflammation and granuloma formation after silica or CNT (22, 44–46). Other key pro-inflammatory cytokines such as type I interferons, IL-12, IL-17, and IL-22 were also dispensable in the development of silica-induced fibrosis (21, 22, 47). The non-requirement of inflammation during experimental lung fibrosis was further supported by the fact that different treatments with anti-inflammatory molecules such as steroids, cox- or phosphodiesterase 5-inhibitors strongly reduced inflammation without modifying, however, collagen deposition (42, 43). The disconnection between inflammation and particle toxicity is not limited to fibrosis. In mice, co-exposure to silica and carcinogens from tobacco smoke resulted in rapid tumor growth in the lungs that is attenuated in the absence of leukotriene B4 axis. This mechanism is, however, independent of IL-1 activation, TNF-α production and chemokine release (48). In addition, early inflammatory reactions triggered by asbestos required NLRP3 inflammasome activation and IL-1β release, but this pathway was not critical in the chronic development of asbestos-induced mesothelioma (49). Recent investigations additionally showed that non-steroidal anti-inflammatory molecules do not reduce progression of asbestos-induced mesothelioma in animals (35, 50).

Altogether, these results strongly suggest that other pathophysiological mechanisms unrelated to inflammation are operational after particle exposure. There are now emerging studies from the literature that explain why diseases achieved by particles are not systematically related to inflammation. This review covers existing evidence supporting that immunosuppression represents an alternative pathological pathway in the development of particle-induced disorders. The most relevant studies delineating the immunosuppressive properties of (nano)particles are depicted in the following sections and commented in Table 1.

**Table 1:** Historical progression of immunosuppression in particle toxicology literature: Summary of the most relevant studies supporting immunosuppressive properties of (nano) particles.

**Authors**	**Highlighted observations**	**Particles**	**Source**
Huaux et al. (51)	An experimental study revealing for the first time that lung responses to silica are unexpectedly characterized by the persistent expression of IL-10, a powerful anti-inflammatory cytokine. *IL-10 was additionally detected after CNT exposure in murine models. Patients developing silicosis or asbestosis exhibit elevated lung levels of IL-10*.	silica *CNT*	**Am J Respir Cell Mol Biol** 18:51-9 **1998** *references #* (37, 52, 53)
Ryan et al. (54)	This original study describes the unanticipated anti-inflammatory effects of NP by negatively regulating allergic inflammation. *This immunosuppressive activity was confirmed in several models of inflammation with various nanoparticles*.	fullerene NP *Oxide and carbon NP*	**J Immunol** 179:665-72 **2007** *references #* (16, 55, 56)
Mitchell et al. (57)	In investigating how CNT suppress immune function, this paper was the first and perhaps the most convincing to propose that nanoparticles induce immunosuppression orchestrated by IL-10 and TGF-β.	CNT	**Nat Nanotechnol** 4:451-6 **2009**
Lo Re et al. (58)	This paper was the first indicating that silica-induced lung fibrosis results from TGF-β-producing regulatory T lymphocytes (T regs). *NP also promote a selective expansion of T regs. The pathological role of T regs was confirmed in silicosis and mesothelioma*.	silica *polystyrene NP*	**Am J Respier Crit Care Med** 184:1270-81 **2011** *references #* (59–61)
Shvedova et al. (62)	This manuscript elegantly demonstrated that TGF-β-expressing MDSC (Myeloid Derived Suppressive Cells) are crucial for tumor development associated to CNT. *MDSC are also implicated in silica-induced lung fibrosis and mesothelioma*.	CNT *silica*	**Small** 243:320-30 **2013** *references #* (63, 64)
Murthy et al. (53)	A comprehensive study showing that asbestos preferentially polarized M2 macrophages during asbestosis in animal and human. *Other particles promoted accumulation of M2 macrophages*.	asbestos *silica and CNT*	**FASEB** 29:3527-36 **2015** *reference #* (65, 66)
Chen et al. (37)	This paper indicated that silicosis and silica-induced lung responses (see related reference #101) are associated with immunosuppressive IL-10-producing B lymphocytes (B regs).	silica	**Front Immunol** 8:110 **2017**

*CNT, carbon nanotubes; NP, nanoparticles*.

## Involvement of the anti-inflammatory and immunosuppressive cytokines TGF-β and IL-10

Indubitably, TGF-β is associated with the development of responses to particles in human and animal. Elevated TGF-β expression has been observed in patients with asbestosis or silicosis (67–71). In a variety of animal models, TGF-β activation has been demonstrated following exposure to silica, asbestos, or CNT (57, 63, 72, 73). After particles exposure, TGF-β contributes to fibroproliferative reorganization of organ tissue by orchestrating myofibroblast differentiation, collagen overproduction, and scar formation (74, 75). Previous studies have demonstrated that treatment with TGF-β inhibitors decreased lung fibrosis in animals exposed to silica (76–78). Additionally, TGF-β contributes to generate a favorable microenvironment for tumor growth and metastasis in mesothelioma, assigning this cytokine as dominant during the development of cancer induced by particles (79). It is noteworthy that the main function of TGF-β is immunosuppression since TGF-β1-deficient mice die from uncontrolled systemic inflammation and autoreactive T lymphocyte accumulation (80). The pivotal role of TGF-β argues for the development of other mechanisms than inflammation after particle exposure and supports that master immunosuppressive cytokines are also linked to particle-related diseases.

**Figure 2:**
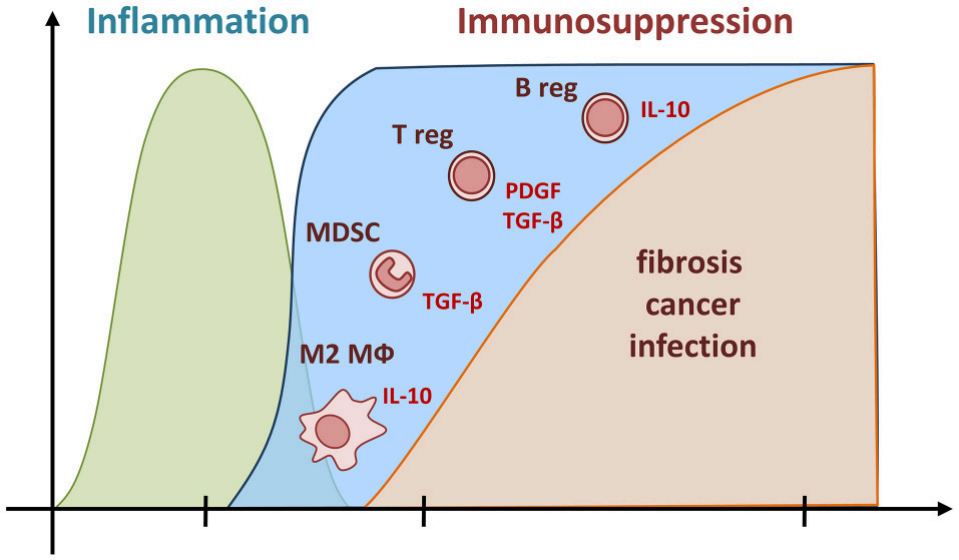
Pathological functions of persistent immunosuppressive cells and mediators during long-term responses to particles. Unresolved Immunosuppression (in blue) represents an alternative event during the responses to particles. According to this new pathological pathway, fibrogenesis, and carcinogenesis are governed by a persistent accumulation of immunosuppressive myeloid (M2 and MDSC) and lymphoid (T and B regs) cells and a sustained production of their related cytokines (IL-10 and TGF-β). These immunoregulatory components limit both the recruitment of inflammatory cells and the activity of pro-inflammatory mediators (in green). The high amount of immunosuppressive cytokines produced can, in addition to their anti-inflammatory action, also act as profibrotic mediators, conceivably by stimulating mesenchymal cells to overproduce collagenase inhibitors and ultimately matrix elements under non-inflammatory conditions. The persistence of immunosuppressive cells and mediators is also incriminated in carcinogenesis and infection by preventing host immune responses directed against transformed cells and microorganisms.

Because the anti-inflammatory cytokine IL-10 was effective in the regulation of inflammation, several authors have suggested that this cytokine could also be active in controlling inflammation in the early and late responses to mineral particles and thus plays a beneficial role based on the inflammatory paradigm (81–83). However, several lines of evidence have demonstrated that IL-10 (as TGF-β) possesses deleterious and pro-fibrotic functions after particle exposure. First, lung expression of IL-10 was intimately associated to the development of particle-induced fibrosis and cancer and could explain the absence of chronic and progressive inflammation in human and mouse models (37, 53, 57, 84–88). Additional data supports that IL-10 participates in the extension of fibrosis since targeted overexpression of IL-10 in the murine airways caused, by itself, collagen deposition, accumulation of mesenchymal cells and airway remodeling with fibrosis (89, 90). Subsequently, it has been demonstrated that IL-10 limits inflammatory response but promotes fibrotic reaction. Indeed, mice deficient for IL-10 developed exaggerated acute inflammation but limited lung fibrosis in response to silica (51, 91). Conversely, overexpression of IL-10 in the lung, by using an adenovirus coding for this cytokine, limited the inflammatory process induced by silica, but increased the fibrotic response (92). It is now admitted that this potent immunosuppressive cytokine has direct effects on fibroblasts and matrix protein release (93). In addition, IL-10 induces the expression of pro-fibrotic mediators (i.e., TGF-β) by macrophages and limits the synthesis of anti-fibrotic mediators (i.e., prostaglandin E2) by epithelial cells (91). The deleterious effect of IL-10 is not limited to fibrosis development but is also implicated in the carcinogenic process resulting from particles. Recent results showed that asbestos exposure restricts effector T lymphocyte activation and impairs anti-tumor immunity through IL-10 release (69, 94). IL-10-expressing macrophages increased mesothelioma cell proliferation and resistance to treatment (85, 95). Collectively, these data additionally challenge the concept that only pro-inflammatory mediators and inflammation lead to fibrosis and cancer and conclusively demonstrate a key role of persistent immunosuppressive mediators in the development of particle-related disorders in the absence of inflammation (Figure 2).

## Contribution of regulatory T and B lymphocytes in response to particles

Based on the immunosuppressive profile of silica-treated mice, the role of regulatory T lymphocytes (T regs) in the development of lung responses to particles was investigated. This specialized thymus-derived sub-population of Th lymphocytes acts to suppress immune responses, thereby maintaining homeostasis and self-tolerance. They mediate immunosuppression by producing the immunoregulatory cytokines IL-10 and TGF-β (96). Recent results indicated that T regs are progressively and specifically accumulated in lung tissue of mice treated with silica where they promote pulmonary immunosuppression and fibrosis (58, 60) (Figure 2). This hallmark of immunological tolerance and immunosuppressive environment was observed with other particles, detected locally and systemically, and induced by the Wnt/B-catenin pathway (57, 59, 97, 98). Lung T regs directly stimulated fibroblast proliferation and collagen deposition after transfer in non-treated mice (58). The direct profibrotic effects of T regs were reduced by inhibiting the PDGF-B/TGF-β signaling pathway, demonstrating that T regs increase tissue fibroblast numbers, and consequently, amplify collagen deposition. *In vitro* and *in vivo* studies additionally showed that mesotheliomagenic fibers such as asbestos directly enhance the immunosuppressive function of T regs and their capacity to release TGF-β and IL-10 (61, 94). In addition, the marked presence of T regs have been detected during lung carcinogenesis after silica or asbestos exposure (99, 100). From these last studies, it has been concluded that the effect of particles on T regs accumulation and function induces a maintained tolerant microenvironment counteracting anti-tumor host innate and adaptive immunity and favoring tumoral cell evasion and the occurrence of particle-induced tumors.

More recently, Chen and colleagues have identified an additional immunosuppressive subpopulation of B lymphocytes recruited in response to silica in human and animal (37, 101) (Figure 2). Regulatory B-lymphocytes (B regs) are immunosuppressive cells releasing IL-10 and TGF-β that support immunological tolerance and suppress immunopathology by limiting the expansion of inflammatory T cells. B regs are defined as the immunosuppressive counterpart of antibody-producing B2 lymphocytes (or conventional B cells) (102). The regulatory B-cell subpopulation has been reported to be recruited to the tumor aggregates and thereby promotes carcinogenesis by attenuating anti-tumor immune responses (103). Their pro-fibrotic functions have been attributed to their capacity to release IL-10 in silica-treated mice (101). As observed in IL-10-deficient mice, reduction of fibrosis in B reg-depleted mice was associated with an increased neutrophilic inflammation (101). Interestingly, IL-10-producing B regs convert T eff into T regs thus increasing immunological tolerance after particle exposure (104). The observation that immunosuppressive T and B-lymphocytes persistently populate damaged tissue strongly suggests that these cells are crucial immune components explaining the development of fibrotic and carcinogenic responses to particles in absence of substantial inflammation.

## Critical roles of immunosuppressive myeloid cells

Infiltrated macrophages in cancer or asthma acquire a particular phenotype called M2, which provides an immunosuppressive microenvironment for tumor growth and allergic tolerance (105, 106). Recent studies also revealed a preferential engagement of immunosuppressive M2 macrophages after inhalation of particles (Figure 2). There is an increased predominance of M2-polarized macrophages in patients developing asbestosis (53). In animal, M2-related mediators such as Arginase 1, crystallizable protein YM-1 or IL-10 were all increased in response to reactive particles such as silica, ultrafine amorphous silica and CNT, appointing M2-polarized macrophages as pivotal (53, 65, 66, 107–109). Authors have recently incriminated oxidized phospholipids, IL-4 or IL-13 in the polarization of M2 macrophages that display enhanced production of TGF-β. Evidence also showed that this last cytokine explains the deleterious activities of M2 macrophages after particle treatment (43, 53, 110–112). These results therefore argue that the continuous accumulation of M2 macrophages and their products, initially for counteracting the effects of inflammatory M1 macrophages, participates in the establishment of the fibrogenic and mesotheliomagenic lesions generated by particles.

Myeloid-derived suppressor cells (MDSC) represent a heterogeneous population of immature neutrophilic and monocytic myeloid cells that displays potent immunosuppressive activities and inhibits immune effector cell functions in diverse chronic pathologies such as cancer (113). The accumulation of immature and immunosuppressive myeloid cells is also a central event during dust-induced fibrosis and cancer with predominant immunosuppression (Figure 2). Indeed, recent investigations have elegantly demonstrated that CNT or asbestos exposure induces a robust accumulation of MDSC that actively release TGF-β and promote cancer progression in mice (62, 64, 114). Fibrotic responses to silica also comprise an accumulation of monocytic MDSC that possess the intrinsic capacity to promote release of the collagenase inhibitor TIMP-1 by fibroblasts. The subsequent elaboration of a non-degrading matrix environment was attributed to monocytic MDSC-derived TGF-β (63). These observations newly indicate that immunoregulatory M2 macrophages and MDSC, along with T and B regs, are implicated in fibrogenesis and carcinogenesis, especially when these cells are continuously accumulated and when inflammatory activity is limited (Figure 2).

## Nanoparticles induce immunosuppression

The emerging concept that particles orchestrate the establishment of immunosuppressive responses is consolidated by several studies exploring the impact of nanoparticles on the immune system. As mentioned above for micrometric particles, the major concern in the field of nanotoxicology is historically related toward the capacity of nanoparticles to induce inflammation and immunostimulation (115). Nevertheless, this exclusive view has been revisited and recent attention has been given to anti-inflammatory and immunosuppressive properties of these particles (55). There is, indeed, a growing body of evidence showing that nanoparticles also display strong immunosuppressive effects (56). In 2007, Ryan and colleagues were the first to report that fullerene nanoparticles prevented mast-cell related diseases such as asthma, arthritis, and sclerosis (54). Other pioneer studies unexpectedly discovered that dendrimer nanoparticles improved disease scores in animal models of granulomatous, autoimmune, and edema diseases (116). Another example illustrating the immunosuppressive properties of nanoparticles is the study by Rajan (117). Liposomal nanoparticles possess the ability to elicit an immunosuppressive cell environment that subsequently suppress anti-tumor immunity and favor tumor progression.

The particles inducing immunosuppression mainly include metal (e.g., gold, silver, iron oxide, cerium oxide, and zinc oxide) and carbon (CNT, fullerenes) nanoparticles (56). The immunosuppressive effects of nanoparticles also depend on their physicochemical properties. For instance, small nanoparticles are more immunosuppressive than large particles (116, 118). The exact physicochemical determinants accountable for nanoparticle-induced immunosuppressive activities remain, however, unclear and require additional investigations. There is also a crucial need to determine whether molecules enrobing particles (e.g., LPS) are important for the immunosuppressive activities of (nano)particles (115).

Interestingly, research in nanotoxicology elucidated different molecular mechanisms explaining how particles promote immunosuppression. These mechanisms involve direct interference of nanoparticles with TLR-, NFκB-, or STAT1-signaling pathways (55, 119). Iron nanoparticles specifically alter IL-1β processing by preventing inflammasome assembly (120). A direct inhibitory interaction between gold nanoparticles and inflammatory cytokines have been also observed from the study of Sumbayev (118). Carbon and cerium nanomaterials exhibit potent free radical-scavenger properties and attenuate oxidant molecule production by particle-activated immune cells. These findings confer to these nanoparticles important antioxidant properties that suppress oxidative stress and inflammatory responses *in vitro* and *in vivo* (119, 121, 122).

Nanoparticles strongly and directly polarize the immune system and establish a preferentially immunosuppressive environment. CNT induce the expression of TGF-β and IL-10 (57) as observed for micrometric silica (see above). Nanoparticles also facilitate polarization of immunosuppressive T regs and anti-inflammatory Th2 lymphocytes (98, 123, 124). Mechanistic studies investigating the effects of nanoparticles on T-lymphocyte immunosuppressive lineage commitment revealed that nanoparticles (carbon, grapheme, and iron) directly interfere with autophagy, ROS production, NFκB-nuclear translocation or antigen processing by dendritic cells (124–126). While nanoparticles mainly operated through DC and T cells, they additionally target macrophages and myeloid cells. Liposomes reduced inflammatory functions of macrophages and increased anti-inflammatory mediator expression in myeloid cells (117).

The exact adverse effects of nanoparticle-induced unresolved immunosuppression are not yet delineated but fibrosis development caused by CNT or cerium has been linked to the immunosuppressive cytokines TGF-β and IL-10 (52, 72, 127, 128). Altered host resistance to infection and cancer represents another possible pathophysiological consequence of maintained immunosuppression. Administration of titanium oxide nanoparticles or liposomes inhibited lymphocyte- and macrophage-related immune responses and increased susceptibility to tumor growth (117, 129). Augmented viral and bacterial infections have also been observed after exposure to carbon nanoparticles (130, 131).

## Mutual roles of inflammation and immunosuppression in responses to particles

**Figure 3:**
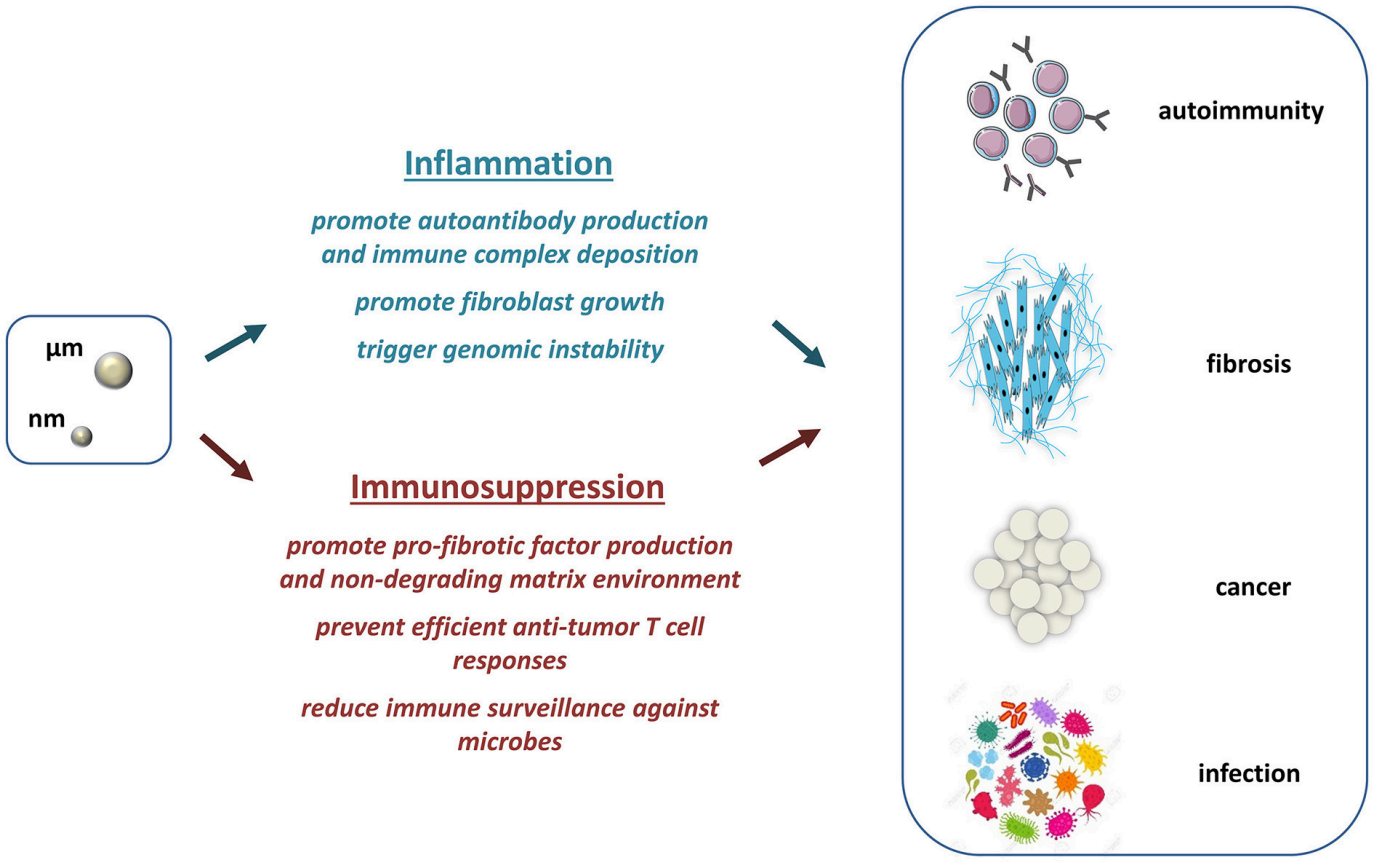
Mutual roles of inflammatory and immunosuppressive responses in particle-induced chronic diseases. The classical scenario explaining the emergence of autoimmune diseases, fibrosis, and cancer after dust exposure mainly relies on predominant and persistent inflammation (blue). Inflammatory responses directly promote fibroblast growth and uncontrolled matrix deposition, trigger genomic instability, and cell transformation, and support autoantibody and immune complex production. An additional paradigm is now offered and comprises predominant and unresolved immunosuppression in response to particles (red). This pathway relies upon a sustained accumulation of immunosuppressive components that include mediators implicated in fibroblast activation, tumor cell and microbe evasion. The exclusive or simultaneous presence of immunoregulatory and immunostimulatory mechanisms can explain the diversity of immune responses and pathologies existing in exposed human or animal.

Taken together, the studies described above and documented in Table 1 support an additional and alternative pathological pathway, which is operative during tissue responses to particles. This new paradigm proposes that fibrogenesis, carcinogenesis, and infections can develop from persistent immunosuppressive developments induced by micro- or nano-particles under non-inflammatory conditions (Figure 3, below). Unresolved immunosuppression participates in the development of fibrosis by activating profibrotic factor release and inhibiting matrix degradation. The immunosuppressive milieu can also favor cancer and infection progression by facilitating tumor and microbial escape from immune surveillance.

This new pathological avenue does not overshadow the relevance of the inflammatory scenario. Indeed, the compelling findings accumulated over the years indubitably support that uncontrolled fibroblast growth and genomic instability rely upon predominant inflammation, especially when immunosuppressive activity is lacking (Figure 3, above). The emergence of immune complex deposition and autoimmune disorders is additionally associated to persistent inflammation in animal or human exposed to particles. The fact that particle-related autoimmune diseases are not mechanistically covered by sustained immunosuppression indicates that inflammation remains a crucial event in response to particles. Importantly, the inflammatory process governs the early accumulation of immunosuppressive cells and mediators by activating several anti-inflammatory signaling pathways (e.g., IL-10) mediated predominantly by NFκB (132). This notion indicates that inflammation can predispose to a developing immunosuppressive environment and that inflammation and immunosuppression are functionally linked.

Altogether, these observations clearly argue for a more complicated mechanism in particle toxicology, wherein both types of immune responses (persistent inflammation and immunosuppression) could promote chronic diseases after particle inhalation. The mutual roles of inflammation and immunosuppression well explain the diversity of particle-induced immune polarization and pathologies (Figure 3). Interestingly, these apparently opposite processes are not systematically exclusive and may be, in contrast, concomitant. The view that uncontrolled immunoregulatory and immunostimulatory responses can co-exist is supported by recent human and animal studies showing mixed inflammatory and immunosuppressive elements (e.g., elevated levels of TNF-α, IL-1β, IL-10, and TGF-β) during fibrogenesis and carcinogenesis induced by particles (6, 133, 134).

## Toxicological and clinical perspectives

It remains, however, to learn how to extrapolate and how to benefit from these observations in predictive toxicology and translational medicine. Current bioassays testing particle reactivity are exclusively based on the appreciation of inflammatory components and suffer from a lack of specificity (135, 136). These tests could be enriched by considering the expression of immunoregulatory mediators or signatures. For instance, release of TGF-β and IL-10 and subsequent abrogation of IL-1 and TNF-α production by treated macrophages could be used to identify reactive particles as proposed by the team of Boraschi and colleagues (137). Furthermore, blockade of inflammasome machinery or antigen-presenting capacity of macrophages and DC could be considered as clear sign of immunosuppressive responses. Inhibition of inflammatory pathologies in animal models could also reflect ability of particles to trigger immunosuppression (56).

In recent years, the increase of anti-tumor responses obtained in clinic trials by blocking the immunosuppressive environment of tumors have revolutionized cancer treatment. Monoclonal antibody-based immunotherapies targeting TGF-β and IL-10 or T cell immune checkpoint receptors (PD-1 or CTLA4) are now available and confer long-term benefit for patients (138–140). Additional experimental and clinical studies are necessary to determine whether this therapeutic option inhibiting immunosuppressive responses is also promising for the treatment of patients affected by particle-associated disorders.

The existence of at least two different pathogenic routes may explain why existing therapies are controversial and disappointing with a strong differential effect in specific subgroups of patients (141, 142). The opportunity to distinguish the prevailing immune response or profile (inflammation and immunosuppression) existing in patients should also help the clinician to categorize patients, assess disease progression and select the most appropriate therapy to inhibit key effector cells and mediators of inflammation (steroids) and/or immunosuppression (immunotherapy). This possibility has been already observed in available animal models. Silica-induced lung fibrosis in rats (associated with chronic inflammation) can be reverted by anti-inflammatory treatment while in mice (developing preferentially unresolved immunosuppressive responses), silicosis is resistant to anti-inflammatory therapy but prevented by T regs neutralization (43, 60). The enthusiasm for the discovery of novel drug targets to treat particle-related diseases should be tempered as inflammation and immunosuppression are extremely imbricated with each other, leading to the fact that inhibition of one response may be compensated by the extension of the other (22, 58).

## Author contributions

The author confirms being the sole contributor of this work and has approved it for publication.

### Conflict of interest statement

The author declares that the research was conducted in the absence of any commercial or financial relationships that could be construed as a potential conflict of interest.

## Abbreviations

T or B regs: Regulatory T or B lymphocytes
MDSC: Myeloid Derived Suppressive Cells
M2 macrophages: Alternatively differentiated macrophages
Th: CD4^+^ T helper lymphocytes
γδ T cells: gamma and delta TCR chain positive T lymphocytes
IFN: Interferon
T eff: Effector T lymphocytes
CNT: Carbon nanotubes
